# The impact of PCR duplication on RNAseq data generated using NovaSeq 6000, NovaSeq X, AVITI, and G4 sequencers

**DOI:** 10.1186/s13059-025-03613-7

**Published:** 2025-05-28

**Authors:** Natalia Zajac, Ioannis S. Vlachos, Sija Sajibu, Lennart Opitz, Shuoshuo Wang, Sridar V. Chittur, Christopher E. Mason, Kevin L. Knudtson, John M. Ashton, Hubert Rehrauer, Catharine Aquino

**Affiliations:** 1 Functional Genomics Center Zurich, ETH Zurich and University of Zurich, Zurich, Switzerland; 2 Spatial Technologies Unit, Department of Pathology, HMS Initiative for RNA Medicine, Beth Israel Deaconess Medical Center, Boston, MA USA; 3 Cancer Center and Cancer Research Institute, Beth Israel Deaconess Medical Center, Boston, MA USA; 4 Broad Institute of MIT and Harvard, Cambridge, MA USA; 5 Center for Functional Genomics, University at Albany, State University of New York, Albany, NY USA; 6 Genomics Division, Iowa Institute of Human Genetics, University of Iowa, Iowa City, IA USA; 7 Genomics Research Center, University of Rochester, Rochester, NY USA; 8 Wilmot Cancer Institute, University of Rochester Medical Center, Rochester, NY USA; 9 Department of Physiology, Biophysics and Systems Biology, Weill Cornell Medicine, New York, NY USA; 10 The WorldQuant Initiative for Quantitative Prediction, New York, NY USA

## Abstract

**Background:**

Transcriptome sequencing (RNA-seq) is a powerful technology for gene expression profiling. Selection of optimal parameters for cDNA library generation is crucial for acquisition of high-quality data. In this study, we investigate the impact of the amount of RNA and the number of PCR cycles used for sample amplification on the rate of PCR duplication and, in consequence, on the RNA-seq data quality.

**Results:**

For broader applicability, we sequenced the data on four short-read sequencing platforms: Illumina NovaSeq 6000, Illumina NovaSeq X, Element Biosciences AVITI, and Singular Genomics G4. The native Illumina libraries were converted for sequencing on AVITI and G4 to assess the effect of library conversion, containing additional PCR cycles. We find that the rate of PCR duplicates depends on the combined effect of RNA input material and the number of PCR cycles used for amplification. For input amounts lower than 125 ng, 34–96% of reads were discarded via deduplication with the percentage increasing with lower input amount and decreasing with increasing PCR cycles. The reduced read diversity for low input amounts leads to fewer genes detected and increased noise in expression counts.

**Conclusions:**

Data generated with each of the four sequencing platforms presents similar associations between starting material amount and the number of PCR cycles on PCR duplicates, a similar number of detected genes, and comparable gene expression profiles.

**Supplementary Information:**

The online version contains supplementary material available at 10.1186/s13059-025-03613-7.

## Background

RNA-seq is a technology applied for quantification of RNA abundance and allows the study of gene regulation and function [1]. Prior to sequencing, extracted RNA is converted to cDNA, and during library construction, it is amplified via polymerase chain reaction (PCR) to enrich properly structured fragments bearing ligated adapters and generate adequate input material for sequencing. PCR amplification is known to introduce bias due to unequal probabilities of amplification of certain molecules, which in turn can impact the accuracy, sensitivity, and precision of transcript quantification [2]. The amount of input material and the number of PCR cycles directly impact the proportion of spurious duplicate reads, but the optimal set of parameters depends on the library complexity and sequencing depth [3].

In RNA-seq, the distinction of amplification-derived duplicates cannot be performed in silico purely by mapping coordinates [4, 5] because it could often result in the removal of a large proportion of biologically relevant information [6]. To this end, Unique Molecular Identifiers (UMIs), which are short (often 5–11 nucleotides) random stretches of oligonucleotides, can be added to the RNA fragments prior to amplification to enable the detection of individual molecules [1–3]. Following sequencing, a computational model accounting for UMI errors can be applied to identify reads with identical alignment coordinates and identical UMI sequences, which are then assumed to be duplicates [3, 7, 8].

The most widely adopted short-read sequencing technology used for RNA sequencing is Illumina’s sequencing by synthesis (SBS) [9]. Alternative short-read technologies have been recently introduced to the market, proposing sequencing approaches which could provide specific improvements in cost, flexibility, sequencing time, and/or throughput [10, 11], including the G4 from Singular Genomics and AVITI from Element Biosciences. The former allows for sequencing of four flow cells in parallel, which improves sequencing efficiency. The latter uses the alternative sequencing by binding (SBB) chemistry that involves the binding of a multivalent fluorescent polymerase substrate by avidity, which is suggested to improve read accuracy and reduce costs [12, 13].

There are currently multiple different vendors providing specific library preparations for all aforementioned technologies. However, the need of sequencing an RNA-seq library generated using Illumina-specific reagents on alternative sequencers is still a common scenario. These libraries contain Illumina-specific adapters, which need to be converted prior to sequencing on a different platform. The conversion protocols include additional PCR steps, which could potentially introduce additional biases such as an increase in the rate of PCR duplicates [10].

In this study, we examine the impact of the amount of RNA input material and the number of amplification cycles on the proportion of PCR duplicates and RNA-seq data quality. Additionally, we systematically evaluate Illumina RNA-seq library performance, quality, and complexity, sequenced across the aforementioned novel HTS instruments after library conversion. For input amounts above the recommended 10 ng (here 15 ng) but below 125 ng, we observe a strong negative correlation between input amount and the proportion of PCR duplicates, but a positive correlation between the number of PCR cycles and the proportion of PCR duplicates. For those input amounts, we show that the highest quality RNA sequencing is obtained using the lowest recommended number of PCR cycles for amplification. We demonstrate the importance of UMIs for those samples for computing gene expression profiles. We also show that the data generated with four different sequencing platforms presents a similar association between starting material amount and the number of PCR cycles with minor differences; the library conversion of Illumina libraries for sequencing on AVITI and G4 resulted in lower abundance of artifactual short reads (mainly primer dimers) but in an increase of PCR duplicate rate for very low input amounts (< 15 ng).

## Results

### Featured datasets

**Fig. 1:**
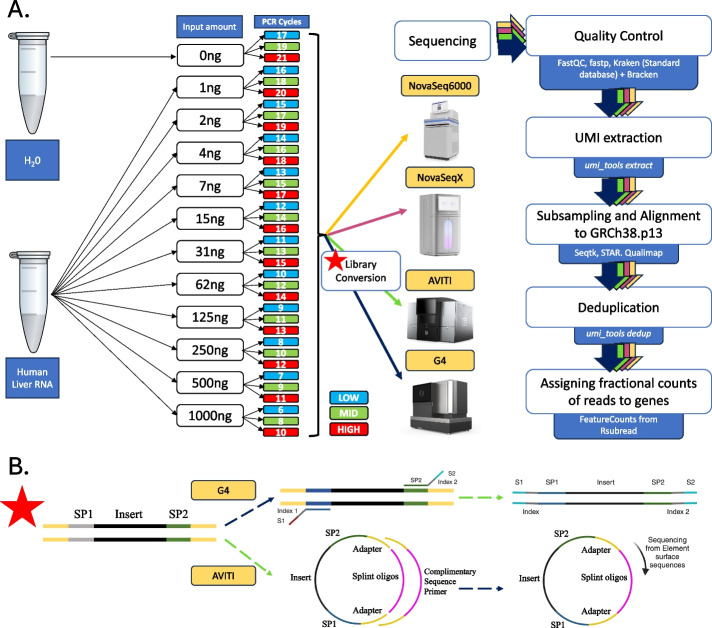
Summarized experimental workflow. **A** Human liver total RNA sample was serially diluted from 1 to 1000 ng and coupled with a negative control of 0 ng of material. Libraries were prepared from each sample using the NEBNext Ultra II Directional RNA Library Prep Kit for Illumina with Unique Dual Index UMI Adapters RNA Set1. cDNA was selectively enriched with PCR containing unique dual indices (UDI) for multiplexing using 3 different numbers of PCR cycles adjusted according to the input amount. The native Illumina libraries were sequenced on two Illumina machines: NovaSeq 6000 and NovaSeq X (images obtained from https://www.illumina.com/, Accessed 22.02.2025). The libraries underwent a conversion and then were also sequenced on AVITI (image obtained from https://www.elementbiosciences.com/products/aviti, Accessed 22.02.2025) and G4 (image obtained from https://singulargenomics.com/g4/, accessed 22.02.2025). **B** The details of the conversion strategies for sequencing of the native Illumina libraries on G4 and AVITI. For G4: the library is amplified in the presence of complementary primers containing G4 indexes and S1 and S2 adaptors which replace the flanking sequences from the kit. For AVITI: the library is circularized with the use of a splint oligo which anneals to the library, followed by rolling amplification to produce the RNA colony forming the basis of avidity sequencing

We generated libraries from human liver RNA at different dilutions (1 ng to 1000 ng) paired with an additional sample of water, serving as negative control (NC). The libraries were generated with NEBNext Ultra II Directional RNA Library Prep Kit for which the minimal supported input amount is 10 ng. Samples were PCR amplified using 3 different levels of amplification, categorized as low/mid/high, with a 2-cycle difference between consecutive levels. The number of cycles was adjusted to the input amount [14] (Fig. 1). For sequencing on AVITI and G4, samples underwent a library conversion (Fig. 1). Following a multi-center setup, the samples were sequenced on four sequencers, including NovaSeq 6000, NovaSeq X, AVITI, and G4, in three different laboratories and sequencing facilities: the Functional Genomics Center Zurich, DNA Technologies and Expression Analysis Core at UC Davis Genome Center, and the Spatial Technologies Unit of the Harvard Medical School Initiative for RNA Medicine at Beth Israel Deaconess Medical Center. For analysis, the samples were subsampled to 2,000,000 reads (Additional File 2: Table S1).

### Raw read quality evaluation

**Fig. 2:**
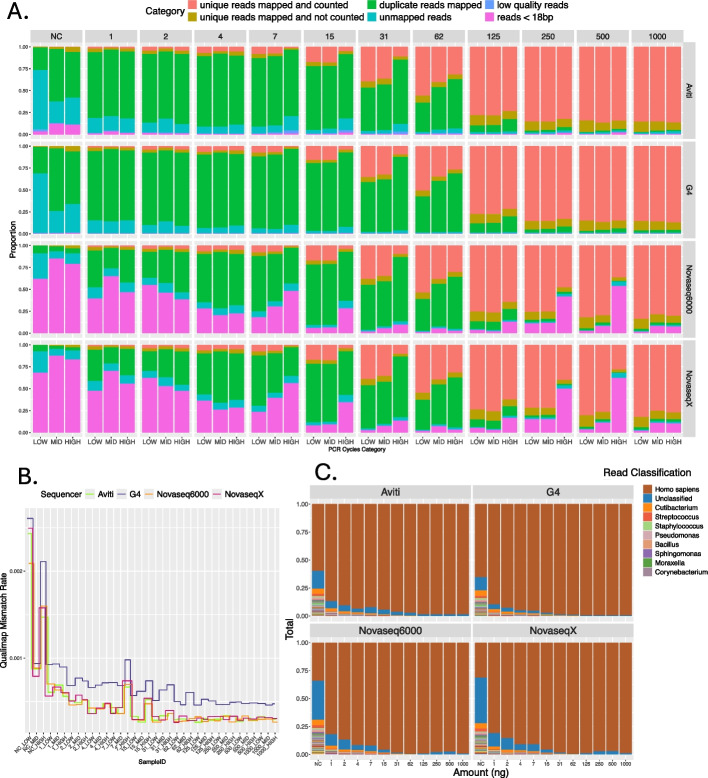
Evaluation of the quality of the RNA-seq data. **A** The classification of total subsampled reads per each sample. The colors indicate the categories: low quality and reads shorter than 18 bp were filtered with fastp, unmapped reads were rejected by the STAR mapper, duplicate reads were removed via deduplication, and only mapped and counted unique reads represent gene expression. Counts used for this figure are attached as Additional File 2: Table S1. **B** Rate of mismatches in raw data mapped to the human genome, measured with Qualimap (v2.2.1). **C** Taxonomic classification, assessed with Kraken and Braken, of the subsampled reads, filtered of low quality and short reads. The data is displayed as per sequencer per input amount (NC - negative control, otherwise input in ng), and the result is an average across all three PCR cycles. The data is sorted by read abundance. The top 10 taxa are displayed in the legend (see Additional File 2: Table S3 for all); the first two categories represent reads mapping to the human genome and reads unclassified by Kraken standard database (05.06.2023). The highest proportion of non-human RNA contamination was found in the negative control samples and samples of input amount below 7 ng

The reads obtained from all four sequencers were of high quality; none of the reads were discarded as low quality (Fig. 2A). The average Phred quality score of reads ranged from 36 to 43, highest for reads sequenced with the AVITI (Additional File 1: Fig. S1). However, the sequencers exhibited variability in sequencing error rate, assessed here as the proportion of mismatches in raw reads mapped to the human genome (GRCh38.p13). The proportion of mismatches for all samples with input amount greater than 1 ng varied between 0.0003 and 0.001. The rate decreased with increasing input amount. For input amounts between 4 and 31 ng there was an elevation of the rate of mismatches for the highest PCR cycle category, suggesting introduction of errors during amplification (Fig. 2B, Additional File 2: Table S2). We observed no difference in mismatch rate between NovaSeq X, NovaSeq 6000, and AVITI, but the data sequenced with G4 had an approximately 50% increase in mismatch rate compared to the other sequencers (Fig. 2B).

The samples for the different sequencers also differed in read composition. For NovaSeq X and NovaSeq 6000, the proportion of artifactual short reads (< 18 bp), inferred to be primer dimers, was higher than for the AVITI and G4 sequencers (Fig. 2A). The percentage ranged from 5.6 to 70.1% for samples of input amounts below 15 ng and from 1.3 to 16.6% for input amounts above 31 ng, with a 10–25% increase from NovaSeq 6000 to the NovaSeq X. Two samples of input amounts 250 ng and 500 ng, amplified using the highest value of PCR cycles and sequenced with the Illumina sequencers, exhibited a fraction of primer dimers comparable to that of the low input amounts (between 41 and 62%), thus creating outliers. Both the AVITI and G4 exhibited low primer-dimer amounts, which can be attributed to the additional library conversion steps and library cleanup. The percentage of primer dimer contamination for the G4 and AVITI ranged from 0.009 to 3.3% across all input amounts (Fig. 2B).

RNA samples often contain a small fraction of microbial contamination, but elevated microbial content can be another reason for considerable data loss during preprocessing and can indicate potential issues in the handling of samples, such as contamination of reagents [15]. We assessed contamination after filtering the data for any short and low-quality reads. For samples between 1 and 15 ng, the microbial contamination ranged from 8 to 1.5%, respectively, across all sequencers. The majority of the bacterial reads mapped to *Cutibacterium*,* Streptococcus*, *Staphylococcus*, and *Pseudomonas.* These top taxa are known to be part of the human skin microbiome [16–18] and thus most likely represent contamination from sample processing. Samples of input amount above 31 ng consisted only of human and unclassified reads, with insignificant traces of microbial content.

**Fig. 3:**
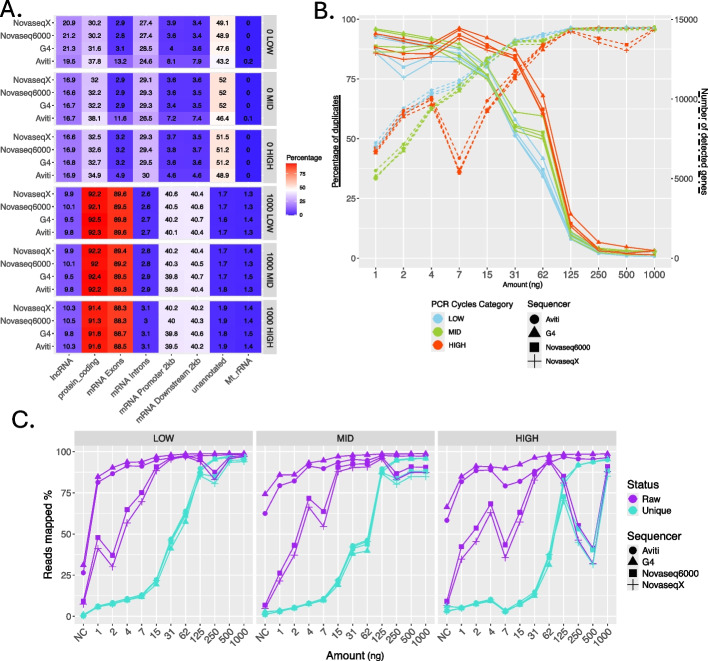
Mapping of the RNA-seq data to the human genome. **A** Proportion of alignments to different features, counting all alignments in the deduplicated bam files (Additional File 2: Table S4). Here we compare the negative control samples (0 ng) to the 1000 ng samples that had the most uniform coverage across the genome. **B** Left: Percentage of duplicates per sample calculated as the ratio of the mapped raw reads to the mapped deduplicated reads. The color indicates the PCR cycle category, and the shape indicates the sequencer. The data is plotted per input amount (NC - negative control, otherwise input in ng). Right: Number of detected genes for each input amount. **C** The percentage of reads per sample mapping to the human genome, calculated as the number of reads out of all the subsampled reads. The data is divided by PCR cycle category, and the color indicates whether the data is before (purple) or after (turquoise) deduplication

Contamination from sample handling can lead to human RNA contamination, potentially introducing biases in downstream analyses. To assess the extent of this contamination, we used negative control samples (0 ng, NC). We anticipated that additional handling during library conversion could further increase contamination levels. Indeed, for the NovaSeq X and NovaSeq 6000, we identified 31–34% of the reads as human RNA (6301–65,838 reads), while for the AVITI and the G4, the 0 ng sample consisted of 60–65% of the reads mapping to human (7532–123,851 reads) (Fig. 2C). We compared the distribution of all alignments from the negative control to that of samples of 1000 ng input and observed that most of the reads from the negative control mapped to lncRNAs (16.6–21.3%), mRNA introns (24.6–30%), and unannotated regions (43–52%). Only 30% to 38% of alignments were to coding regions and only 3–13% belonged to exons (Fig. 3A). Thus, we conclude that the effect of human RNA contamination for all samples on gene expression results was negligible; the unique reads counted into gene expression ranged only from 142 to 2738 of reads for NovaSeq X, NovaSeq 6000, and G4 and between 1530 and 5658 for AVITI.

### Number of artifactual reads depends on a combined effect of input amount and the number of PCR cycles

We observed the percentage of PCR clonal artefacts to decrease with increasing input amount, dropping down to a mean of 3.5% and plateauing at 250 ng (Fig. 3B). Between 82 and 96% of reads were discarded for the amount of 7 ng and between 8 and 18% for the 125 ng input. For between 7 and 125 ng, using the highest recommended value of PCR cycles resulted in the highest proportion of PCR duplicates. Increasing the number of PCR cycles from lowest (low) to intermediate (mid) did not result in a significant increase in the PCR duplicates, except for the input amount of 62 ng, with a shift from 34–42% of duplicates to 50–60% from the low to mid PCR cycle category (Fig. 3B).

Failing to remove PCR duplicates can falsely inflate the perceived mapping rate. For low input amounts, i.e., between 1 and 15 ng, the higher rate of PCR duplicates in libraries sequenced on AVITI and G4 resulted in a higher mapping rate than those sequenced on Illumina sequencers (Fig. 3C). The discrepancy between the sequencers disappeared entirely after removal of duplicates and resulted in only 3–22% of reads being productive. Input amounts above 250 ng yielded the highest mapping rate and the highest proportion of retained reads, and the results were not influenced by the value of PCR cycles used for amplification (Fig. 3C). The proportion of duplicates ranged from 1 to 7% (Fig. 3B) and the percentage of total reads mapped ranged between 85 and 97% (Fig. 3C). For 250 ng and 500 ng samples sequenced with NovaSeq 6000 and NovaSeq X, the number of mapped reads amounted to less than 53% in the highest PCR cycle category and between 80 and 88% in the mid cycle category. The read dropout can be explained by the high percentage of primer dimers in both samples (between 41 and 62%), most noticeably produced when the highest number of PCR cycles was applied (Fig. 2A).

### Number of detected genes is positively correlated with the input amount and can be obscured by the rate of PCR duplicates

The main goal of most RNA-seq analyses is to study the gene expression within a sample or to compare the relative gene expression between samples/groups. For low input amounts, even with the best library protocols, there is a higher chance of loss of information with the loss of input material during sample processing [19]. Additionally, low input amounts require higher amplification for obtaining sufficient material, leading to a higher tendency for highly expressed genes to produce identical fragments, which in turn can lead to a lower probability of sampling the transcripts of lowly expressed genes during sequencing [3]. PCR duplicates create noise that increases the false positive rate, obscuring the number of detected genes and interfering with the absolute and relative quantification of expression [2, 20].

**Fig. 4:**
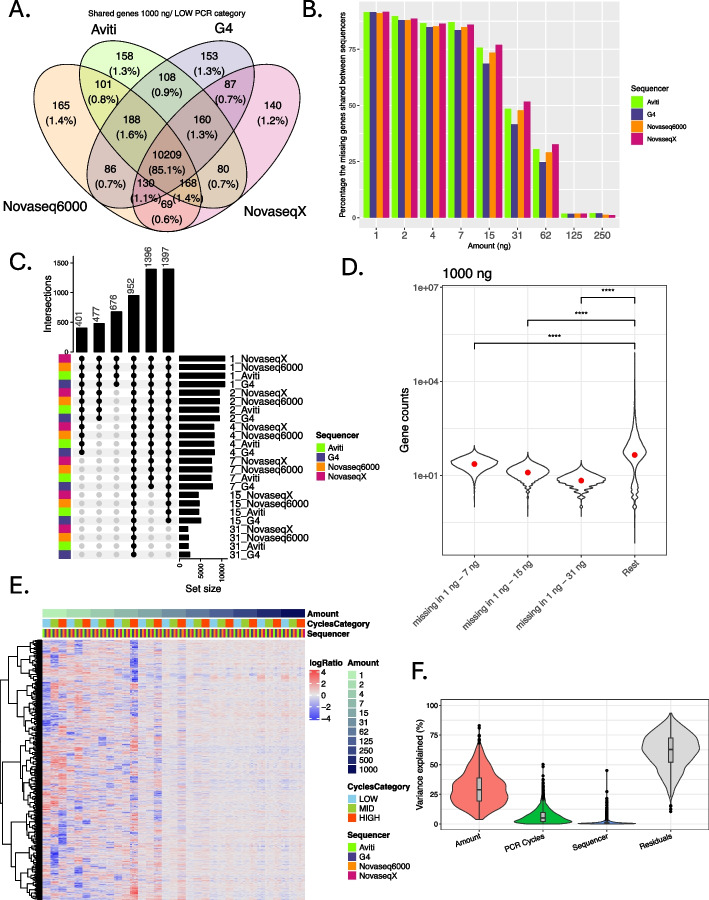
Number of detected genes. **A** The number (and percentage) of detected genes from 1000 ng input amount from the lowest PCR category shared by the four sequencers. All four sequencers shared 85% of all the detected genes and 90% of the genes were detected by at least 3 sequencers. **B** Percentage of missing genes (compared to those detected at 1000 ng, counts > 5) shared across sequencers per input amount. Data from different PCR cycle categories were pooled. **C** Overlap in missing genes across all samples from low input amounts (< 31 ng). Data were pooled across all PCR cycle categories. **D** Gene counts obtained from 1000 ng input amount (pooled across all sequencers and all PCR cycle categories) for genes missing from the three categories based on the three last columns of the upset plot in **C**. Significance of the differences between means was tested with Mann–Whitney *U* test. **E** The normalized, log2 transformed counts centered by row for the 1582 genes found to be expressed across all samples. The columns are ordered by input amount (in ng), sequencer, and PCR cycles category. **F** Percentage of variance explained by the three factors in gene expression differences between samples for the 1582 genes found to be expressed across all samples

We found that the number of detected genes positively correlated with the input amount (Fig. 3B). The number of genes ranged from 5013 at 1 ng to 14,536 at 1000 ng (Additional File 2: Table S1), across all sequencers (Fig. 3B). For input amounts above 125 ng, there was no increase in the number of detected genes (Fig. 3B) and we observed a high congruency in the genes detected from each of the sequencers; across the samples of 125 ng to 1000 ng input amount, amplified with the lowest number of PCR cycles, 90% of all the genes detected were shared by at least 3 sequencers, and 85% were shared by all 4 sequencers (Fig. 4A, Additional File 1: Fig. S2).

For input amounts between 15 and 125 ng, we observed that the highest rate of PCR duplicates generated with the highest recommended number of PCR cycles obscured the possible number of detected genes (Fig. 3B). At 7 ng, around 50% more genes were detected when the data was amplified with the mid or low number of PCR cycles as compared to the high number of PCR cycles. For 62 ng, that difference decreased to only 5% more genes. For input amounts above 125 ng, the number of PCR cycles had no effect, except for two outliers—250 ng and 500 ng—where 13,063 to 13,828 genes were detected in the highest PCR cycle category. The lower number of genes corresponded to the lower percentage of usable reads caused by the contamination with primer dimers (Fig. 2A) and with unclassified contaminants (Fig. 2C).

We observed a notable consistency in the missing genes across sequencers for low input amounts. For 1 ng to 15 ng, 69% to 92% of the genes absent in one sequencer (compared to those detected at 1000 ng, counts > 5) were also missing from the outputs of other sequencers (Fig. 4B). This suggests that gene loss primarily results from transcript loss during the serial dilution, driven by either sampling bias or low transcript expression, rather than by sequencing bias. For input amounts between 31 and 62 ng, the result was much lower (25–52%, Fig. 4B) indicating that higher amounts of starting material result in more random gene loss.

We found 1396, 1397, and 952 genes missing (compared to those detected at 1000 ng, counts > 5) from all 1–7 ng, 1–15 ng, and 1–31 ng samples, respectively, after pooling all 3 PCR categories together. We found those genes to be significantly less expressed at 1000 ng (Mann–Whitney *U* test, *p*.adj < 0.05), indicating the higher probability of capturing lowly expressed genes with increasing input material (Fig. 4D). We did not observe any structural reasons for gene loss—the missing genes had a slightly elevated GC content (mean of 50% vs 52% across all groups, Mann–Whitney, *p*.adj < 0.05) but no differences in length (mean of 2020–2086 bp across all groups, Mann–Whitney, *p*.adj > 0.05) (Additional File 1: Fig. S3). No structural differences were also observed for genes detected by both low and high input amounts (7–15 ng + 250–1000 ng, count > 5) when compared to genes detected only high input amounts (250–100 ng, count > 5), disregarding the PCR cycle differences within each sequencer (Additional File 1: Fig. S4).

### Low input amounts paired with overamplification yield distorted gene counts

**Fig. 5:**
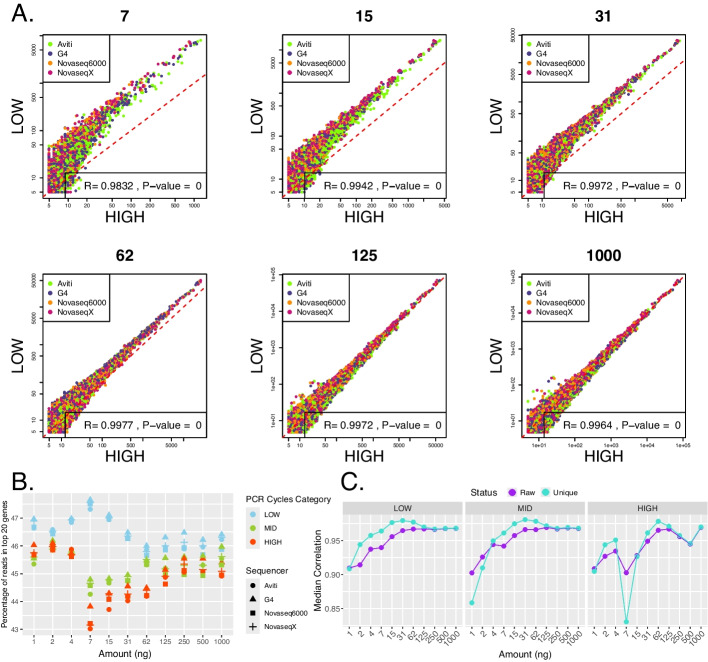
**A** Correlation of gene counts between high and low PCR cycle categories from deduplicated reads per input amount (in ng), with an added background expression of 5. The points are colored by the sequencer and the *x*- and *y*-axes are log-transformed. Pearson’s correlation coefficient (*R*), which determines the strength of a linear correlation (ranging from 0 to 1), is indicated, together with the *p*-value, at the bottom of the plot. One-to-one reference line is added in red. **B** The percentage of total counts captured by the top 20 most highly expressed genes. The points are colored by the PCR Cycles Category and the shape indicates the sequencer. High PCR cycles category captures a lower percentage of total counts in the top 20 highly expressed genes than the low PCR cycles category, most clearly for input amounts between 7 and 62 ng. **C** Consistency between technical replicates: median Spearman correlation coefficient from correlation of gene counts obtained from the different sequencers. The plots are limited to genes shared between all PCR cycle categories and all sequencers for each input amount. The points are colored by the status of the reads, raw = raw reads including duplicates, unique = unique, deduplicated reads

We find that the combination of input amount and the number of PCR cycles had an influence on the relative gene counts obtained for the same 1582 genes detected across all samples from the unique, deduplicated reads (Fig. 4E). Low input amounts of below 31 ng showed a higher degree of deviation in comparison to high input amounts of above 62 ng (Fig. 4E). On average, 30% of variance in gene expression across those genes was explained by the amount of starting material, and only 7% of variance was explained by PCR cycle category (Fig. 4F). High PCR cycle category rendered lower counts for all genes found to be expressed within each input amount than the low PCR cycle category (Fig. 5A); 2–5% less reads were captured for the top 20 expressed genes (Fig. 5B). Gene counts for input amounts above 250 ng from high and low PCR cycle categories showed a linear correlation (Pearson’s correlation, *R* = 0.99, *p* < 0.05), demonstrating the low impact of the number of cycles used for amplification (Fig. 5A, Additional File 1: Fig. S5).

UMIs are widely used in the field of RNA-seq to differentiate biological copies from PCR duplicate reads [3] and, in our study, the samples sequenced on the different sequencing machines could be used to test their efficiency in removal of artefacts. We find that the median correlation between sequencers increases with the increasing input amount for both the raw and the unique reads (Fig. 5C). Below the input of 125 ng, the correlation between the sequencers improves after deduplication of the data, suggesting the importance of the use of UMIs for removal of artefacts, but for input amounts above 250 ng, there is no significant effect. For the input amounts of below 7 ng amplified with the number of cycles corresponding to either mid or high PCR cycle categories, the correlation between the four sequencers actually decreased after deduplication, showing the inconsistency and bias in the obtained expression profile when low input amounts are used, and the library preparation parameters are not optimally adjusted.

## Discussion

RNA sequencing library preparation is highly sample dependent and protocol specific. An important challenge in using the RNA-seq technology is choosing an optimal set of parameters for library and sample preparation and understanding how the variation within the recommended set of parameters impacts the amount of information that can be extracted from the data after sequencing. On top of that, there is currently an impressive plurality of novel short-read sequencing instruments, and those alternatives to Illumina offer a very popular option to utilize processed Illumina libraries following a conversion step. However, there is no published data on its performance and potential downstream effects of the additional polymerase chain reaction (PCR) cycles required for its application.

In this work, firstly we investigated whether the input amount and the number of PCR cycles correlated with the production of PCR duplicates—artifactual reads that have to be discarded from analysis. We used the UMIs for identification of PCR duplicates, additionally assessing the efficiency of one of the most widespread methods for duplicate read removal [3, 21]. We find that for input amounts above 250 ng the rate of PCR duplicates is negligible and varying the number of PCR cycles applied for amplification does not have an effect. We find less than 7% of the reads being identified as duplicates based on the UMI and alignment coordinate. Those discarded reads did not alter the gene expression profile of the samples in a way that would impact downstream analysis.

However, we find a strong impact of the combination of the input amount and the number of PCR cycles on the rate of PCR duplicates when the starting material is below 125 ng. We observe that for the samples of 7 ng to 62 ng, the input amount is strongly negatively correlated with the proportion of PCR duplicates and thus positively correlated with the proportion of recovered, usable reads and the number of detected genes. We observe a strong decrease in data quality when the highest number of PCR cycles is applied—we detect a much higher loss of detected genes, especially genes that are lowly expressed, and a deviation from the estimated gene expression in comparison to the lower value of PCR cycles. Variation in gene expression caused by unevenness of coverage introduced by amplification has already been observed in previous studies [22, 23].

The impact of those two factors, especially the input amount but also the number of PCR cycles, on the rate of PCR duplicates has already been observed in other studies, both in RNA and DNA sequencing [3, 24, 25]. The amount of starting material has been inferred to correlate with the library complexity or the number of distinct molecular species in a library [26]. Library complexity has been shown to have a stronger impact on the rate of PCR duplicates in comparison to amplification noise dependent on the number of PCR cycles [26]. Our study is concordant with that conclusion.

PCR duplicates are more difficult to identify in RNA sequencing in comparison to DNA sequencing, and methods to distinguish them and the importance of their removal have been a topic of research [2, 6, 27]. We find that UMI deduplication is important and effective for reliable removal of the high proportion of PCR duplicates from samples with low amounts of starting material, without removal of valuable biological information. For input amounts below 125 ng, between 34 and 96% of reads were discarded via deduplication. Removal of spurious reads resulted in more comparable gene expression between the different PCR cycles. The highest rate of PCR duplicates and also the highest impact of deduplication was observed for input amounts below 7 ng, for which below 13% of the reads were estimated to be productive, confirming the recommendations of the library protocol suggesting a minimum of 10 ng.

We also investigated the correspondence in the results between the different sequencers. We observe the same patterns of the effect of starting material and the number of PCR cycles on PCR duplicates, a similar number of detected genes and a comparable gene expression. We observe only a few minor differences. Firstly, for input amounts below 15 ng, we observe a higher rate of PCR duplicates in data from AVITI and G4, driven by the additional PCR cycles in the conversion protocols. This highlights the increased importance of using UMIs for deduplication or considering sequencer-specific library kits offered from these providers. Secondly, for input amounts below 15 ng we observe a higher proportion of reads filtered due to length (< 18 bp) in the data from both of the Illumina sequencers, which emphasizes the importance of library cleanup steps for primer dimer removal. Thirdly, for the G4 sequencer we observe an elevated sequencing error rate measured as the number of mismatches in the mapped raw reads. However, we do not see any impact of that on downstream results including the mapping rate, the number of detected genes or gene expression profiles.

One noticeable difference between the sequencers was the presence of contamination by adapter primer dimers. Two samples from NovaSeq 6000 and NovaSeq X with input amounts of 250 ng and 500 ng did not match the high-quality results from the rest of the samples due to a higher proportion of adapter primer dimers. The effect was most prominently visible when the samples were amplified using the highest PCR cycle category. Primer dimers were removed during conversion of the Illumina library to a library suitable for sequencing on AVITI and G4. These samples serve as an example that with an increase in the number of PCR cycles for amplification, the rate of adapter primer dimerization also increases [28]. To avoid wasting sequencing efforts and production of low-quality data, size selection in library preparation could be applied to filter out the contaminating primer dimers. However, one has to note that size selection itself can introduce transcript length bias also resulting in complications in downstream analyses [22].

## Conclusions

Our results clearly demonstrate that a choice of one of the lower RNA input amounts (below 62 ng) in combination with the highest number of PCR cycles used for amplification can lead to a loss of even up to 35% of the expressed genes from RNA sequencing experiments and can cause a surge in the rate of PCR duplicates, creating noise or interference. The most profound effect is the loss of the lowly to moderately expressed transcripts that in turn could be related to specific lowly or moderately expressed metabolic functions [20]. We thus recommend targeting input amounts above the recommended minimum and we advise against overamplification. Additionally, it is clear that the results from the four different sequencing technologies are highly reproducible, and so we conclude that the choice of the sequencer itself will not have an impact on an RNA sequencing study. We envision our research to become a start of the conversation on how the different technologies can be used in different sample contexts.

## Methods

### Library construction

A serial dilution from 1 to 1000 ng Human Liver Total RNA (purchased from ThermoFisher) was prepared to generate the various input samples. The NEBNext Ultra II Directional RNA Library Prep Kit for Illumina with Unique Dual Index UMI Adapters RNA Set1 (NEB, Franklin Lake, NJ, USA) was used in the succeeding steps according to the manufacturer’s instructions. Briefly, total RNA samples (1–1000 ng) were polyA enriched and then fragmented prior to reverse-transcription into double-stranded cDNA. The cDNA samples were end-repaired before ligation of adapters containing UMI. Fragments containing adapters on both ends were selectively enriched with PCR containing unique dual indices (UDI) for multiplexing. Per dilution, 3 different PCR cycles were used. The quality and quantity of the enriched libraries were validated using a TapeStation (Agilent, Santa Clara, CA, USA). The product is a smear with an average fragment size of approximately 260 bp. The libraries were normalized to 10 nM in Tris–Cl 10 mM, pH8.5 with 0.1% Tween 20. As the different dilutions and PCR cycles used resulted in very varied library concentrations, the pooling was simplified by using 5 µl of the libraries produced.

### Next-generation sequencing

#### Illumina NovaSeq 6000 and Illumina NovaSeq X

The pool of Illumina libraries was quantified using a TapeStation (Agilent, Santa Clara, CA, USA) and normalized to a loading concentration specific for the instrument type. For the NovaSeq 6000 (Illumina, Inc, CA, USA), 18 µl of the pooled libraries with a concentration of 0.8 nM was loaded on a lane of a NovaSeq 6000 SP Reagent Kit v1.5 (100 cycles) flow cell for a final loading concentration of 180 pM. For the NovaSeq X (Illumina, Inc, CA, USA), 34 µL of the pooled libraries with a concentration of 0.55 nM were loaded into a lane of a NovaSeq X Series 10B Reagent Kit (300 Cycle) flow cell for a final loading concentration of 110 pM. The pools were sequenced single-end 100 bp on the NovaSeq 6000 and paired-end 150 on the NovaSeq X.

#### Element Biosciences AVITI

The pool of Illumina libraries was prepared for sequencing on the AVITI sequencer (Element Biosciences, San Diego, CA) using the Element Adept Library Compatibility Kit v1.1 (https://go.elementbiosciences.com/adept-workflow-standard-user-guide-ma-00001, Accessed 22.02.2025). This process involves the denaturation, library circularization via ligation to a splint adapter, and exonuclease digestion of non-circularized molecules. Thirty microliters of the Illumina sequencing library pool at a concentration of 16.7 nM were circularized. The resulting circularized library was quantified via qPCR using the standards provided in the compatibility kit and qPCR (SYBR Green PCR Master Mix, Applied Biosystems, Waltham, MA). Twenty-five microliters of the circularized library at a concentration of 3.5 pM were loaded onto the AVITI system and sequenced with an AVITI 2 × 150 Sequencing Kit.

#### Singular Genomics G4

To enable anchoring of clusters on Singular flow cells, custom conversion primers targeting P5 and P7 with Singular specific (S1 and S2) 5′ overhangs were used to retain the original indexes (Adapters and Indices for G4 Sequencing platform, https://singulargenomics.com/wp-content/uploads/2022/10/Adapters-and-Indices-for-G4-600007-087.pdf, Accessed 22.02.2025). For sequencing, 2 mL of custom index primers (1uM) were loaded into the custom primer wells of 300 Cycle reagent cartridges (Lot 2,304,251). The library pool was quantified with a dsDNA HS Assay Kit (Q33230) on a Qubit 4 fluorometer (ThermoFisher) and diluted down to 1 ng/uL with Ambion RNAse-free water, then amplified with Roche KAPA HiFi HotStart ReadyMix in a BioRad C1000 thermal cycler for a total cycle number of 7. Annealing was set for 30 s at 57 °C. Subsequently, PCR product was cleaned up with SPRISelect beads (Beckman-Coulter) and verified on Fragment Analyzer 5200 using Agilent DNF-473 NGS Fragment Kit (1–6000 bp). To determine the optimal loading concentration, 200 pM libraries and the 50 pM PhiX Control were diluted down to perform a titration run with a series of 12 pM, 15 pM, 17 pM, and 20 pM. Final sequencing was performed using 15 pM loading concentration on two separate F2 flow cells (Lot 4,052,120, Serial number OM0075H and #OM0075H) using a read length setup of 8 (i5, index 1): 100 (Read 1) as well as 19 (i7, index 2 and UMI): 100 (Read 2). Noteworthy, on the Singular G4 platform, i5 corresponds to index 1 and i7 corresponds to index 2, instead of reverse complement i7 and i5, respectively, as on the Illumina or AVITI platform.

#### Demultiplexing

The demultiplexing for the G4 and Aviti data was performed with the sgdemux tool (https://github.com/Singular-Genomics/singular-demux). TThe Illumina data were demultiplexed using bcl2fastq v2.20 (https://support.illumina.com/downloads/bcl2fastq-conversion-software-v2-20.html). The UMIs were located within the i7 adapter sequence in either position 1 to 11, as for NovaSeq 6000 and NovaSeq X data, or 2 to 12, as for AVITI and G4 data.

### Data analysis

The quality of the data was assessed using FastQC v0.11.9. For each sequencing technology, the data were subsampled to 2,000,000 reads per sample. For the negative control samples, the maximum number of reads was taken if the number of reads was lower than 2,000,000. For the total number of reads per sample see Additional File 2: Table S1. Even though the sequencing was performed in a paired-end mode using G4, AVITI, and NovaSeq X, only the forward reads were used in the analysis for even comparison with the NovaSeq 6000 sequencing, which was performed in single-end mode. The subsampled reads were processed using fastp v0.23.4, which involved trimming of Illumina adapters and filtering of reads below 18 bp in length [29]. Reads of length below 18 bp were considered primer dimers. The produced reads were 100 bp long for G4 and AVITI, 101 bp long for NovaSeq 6000, and 151 bp long for the NovaSeq X, subsequently also trimmed to 101 bp. The number of reads filtered due to length was used for estimation of the proportion of primer dimers in each sample. Subsequently, the level of contamination in the filtered reads was estimated with Kraken2 v2.0.9 using the Standard database (05.06.2023) followed by Bracken v2.8 for abundance estimation of human and non-human reads [30–33]. Abundance estimation was run at a genus level. The final abundance was re-estimated after including reads unclassified by Kraken.

The data were then processed using UMI-tools and STAR [7, 34]. First, the UMIs were extracted from the reads and inserted into the read names using the *umi_tools extract* option. The reads were then mapped with STAR within SUSHI [35] in a 1-pass mode to GRCh38.p13 reference (Gencode 42 release), allowing a maximum of 10 mismatches between the read and the reference and a maximum of 50 multiple alignments per read, outputting alignments only if the number of matched bases was higher than 30 bp. Deduplication was performed with *umi_tools dedup*, using the directional method for identifying clusters of connected UMIs and an edit distance threshold of 1. The number of aligned reads was obtained by computing the number of primary alignments using samtools flagstat (samtools v1.17). The mapping quality (mismatch rate and GC content) of the raw and deduplicated data was assessed with Qualimap v2.2.1 [36, 37]. FeatureCounts from the Rsubread v2.14.1 Bioconductor package [38] was employed for assigning mapped sequencing reads to genomic features, taking into account the multi-overlapping and the multi-mapping reads, counting each alignment fractionally. The R package stats v4.3.0 was used to perform Pearson’s correlation analysis of gene expression. Comparison of counts for genes expressed across all samples was performed on counts that were normalized, using the geometric mean scaling normalization method from EdgeR, log2 transformed and centered per gene (divided by the mean). The contribution of the factors such as the input amount, PCR cycles category, and type of sequencer to the amount of variance in gene expression was quantified using a linear (mixed) model from the R package variancePartition (v1.34.0).

## Supplementary Information

Additional File 1: Contains all supplementary figures from S1 to S5.

Additional File 2: Contains all supplementary tables from S1 to S4.

Additional File 3: Contains the review history.

## Data Availability

The raw and processed data is available on the Gene Expression Omnibus database under a bioproject accession: PRJNA1086933 and GEO accession: GSE261432 [40]. The code used for data analysis and creating figures is available on github: https://github.com/zajacn/PCR_Duplicates_RNA [41] (archived source code: 10.5281/zenodo.15295076[42]).
