# Supplementary material for: The impact of PCR duplication on RNAseq data generated using NovaSeq 6000, NovaSeq X, AVITI, and G4 sequencers
Source: Genome Biol. 2025 May 28;26:145. doi: 10.1186/s13059-025-03613-7 (PMC12117910; doi:10.1186/s13059-025-03613-7)
Supplement: Supplementary file 1 — Additional File 1: Contains all supplementary figures from S1 to S5. [file 13059_2025_3613_MOESM1_ESM.docx]

# Supplementary Figures for “The impact of PCR duplication on RNAseq data generated using NovaSeq 6000, NovaSeq X, AVITI and G4 sequencers.”

Fig. S1. Distribution of phred quality scores across all unsubsampled reads for all four sequencers, obtained from MultiQC reports.

Fig. S2. The number (and percentage) of detected genes from 500 ng, 250 ng and 125 ng input amounts from the lowest PCR category shared by the four sequencers. All four sequencers shared approximately 85% of all the detected genes and 90% of the genes were detected by at least 3 sequencers.

Fig. S3. GC content and gene length for genes missing from the three categories outlined in c.): missing from 1-7ng, missing from 1-15ng and missing from 1-31ng. Red line indicates 50% of GC content or 2000bp gene length respectively. Significance of the differences between means was tested with Mann–Whitney U test.

Fig. S4. The length and GC content of genes detected only in high input amounts (from 250 to 1000 ng) or by both high and low input amount (between 7 and 31 ng and between 250 and 1000 ng). The information about the number of PCR cycles for amplification is disregarded. a.) Gene length of the two categories of genes per sequencer b.) GC content of genes from the two categories per sequencer and c) summary table of the number, mean length and mean GC content of genes detected by both low and high input amouts, only by low or only by high input amounts.

Fig. S5. Correlation of gene counts between high and low PCR cycle categories from deduplicated reads, with an added background expression of 5. The points are coloured by the sequencer and the x and y axes are log-transformed. Pearson’s correlation coefficient (R), which determines the strength of a linear correlation (ranging from 0 to 1), is indicated, together with the p-value, at the bottom of the plot. One-to-one reference line is added in red. The exceptions were the samples of 250 ng and 500 ng from NovaSeq X and NovaSeq 6000 that exhibited a deviation in counts between the High and the Low PCR cycles categories, elucidated by the low proportion of mapped reads for those two samples (see main text for more information).


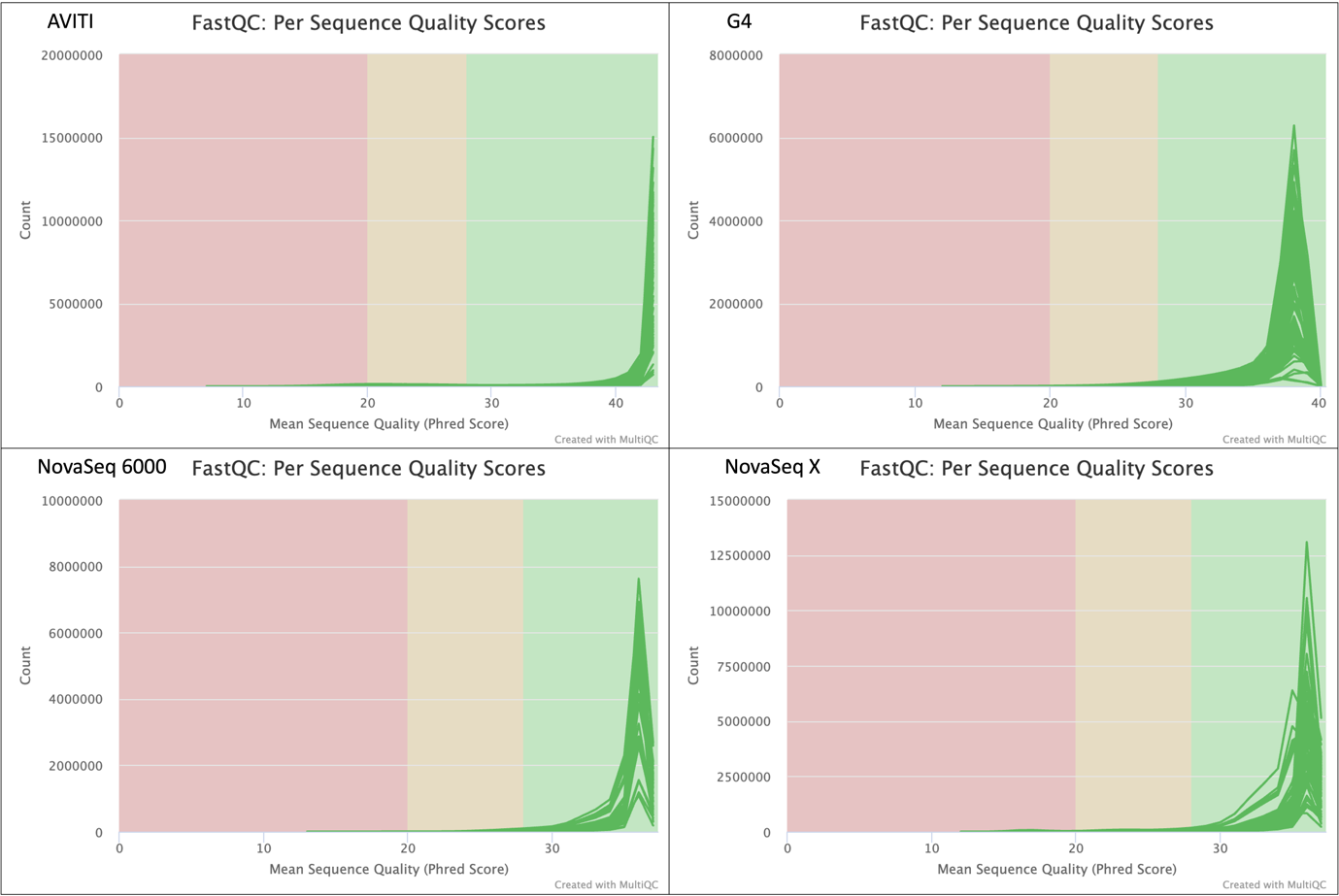


Fig. S1

Fig. S2

Fig. S3


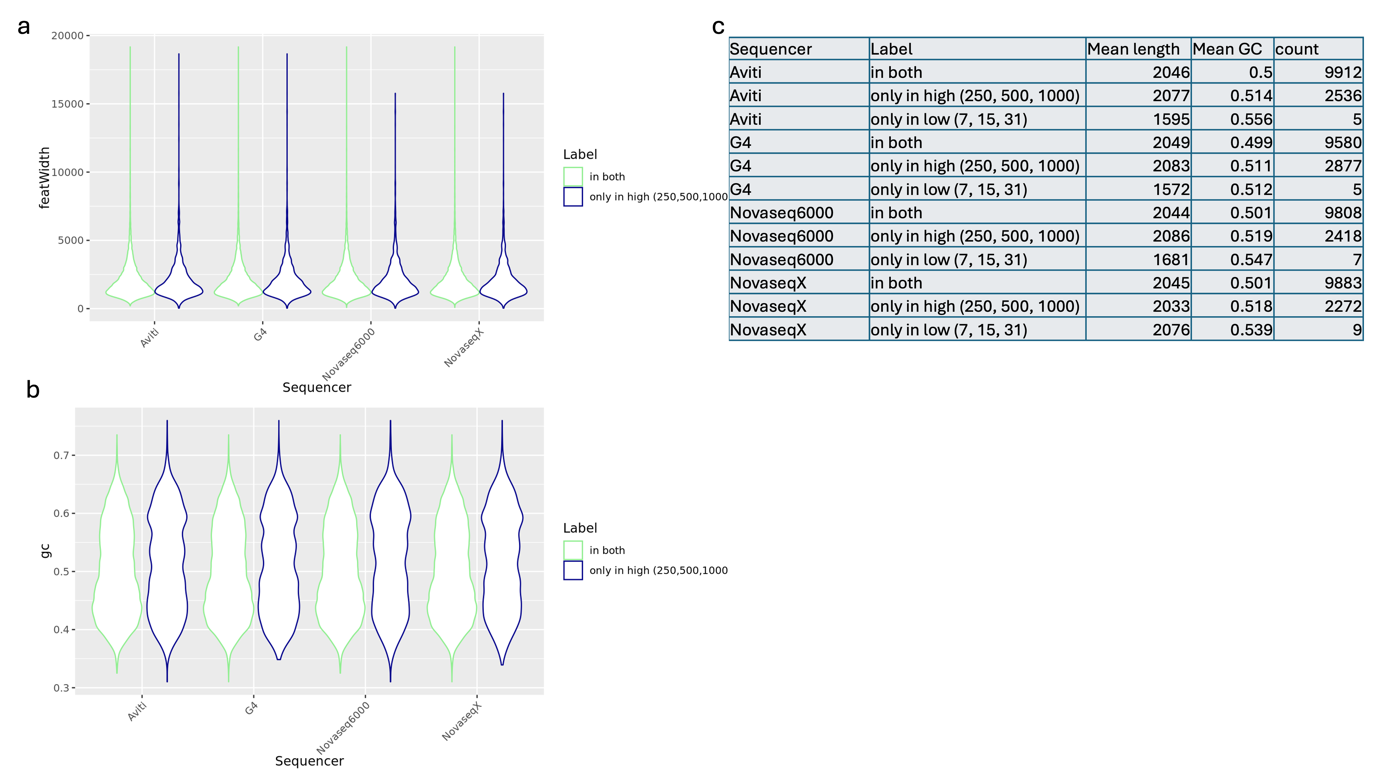


Fig. S4

Fig. S5
