# Supplementary material for: The impact of PCR duplication on RNAseq data generated using NovaSeq 6000, NovaSeq X, AVITI, and G4 sequencers
Source: Genome Biol. 2025 May 28;26:145. doi: 10.1186/s13059-025-03613-7 (PMC12117910; doi:10.1186/s13059-025-03613-7)
Supplement: Supplementary file 3 — Additional File 3: Contains the review history. [file 13059_2025_3613_MOESM3_ESM.docx]

**1^st^ round**

**Reviewer 1**

In this study, Zajac et al. look to investigate the technical impacts of low amounts of RNA input and higher amounts of PCR cycles on RNAseq analysis of gene expression across a number of sequencing platforms. The authors claim that lower amounts of input material result in higher duplicate levels and that UMIs result in more comparable gene expression data. The authors suggest only minimal impact from the choice of sequencing platform.
The question asked by the authors is an important one as many researchers assume these critical parameters are immaterial to their analyses and can focus major scientific conclusions on what were technical artifacts. The authors do observe significant sequencing saturation of samples with lower input amounts and observe negative impacts of additional amplification. However, the manuscript, as written, is extremely challenging to read and focuses heavily on minor points, hiding the critical results. The figures are poorly designed, do not clearly demonstrate the experiments done, and do not highlight the key results. The study also appears to be highly underpowered with possible technical artifacts visible in the results. For a paper that highlights these artifacts, having an underpowered analysis is not acceptable. Thus, while the issues highlighted by the authors are critical, major revisions should be done prior to acceptance.

MajorIssues
* A number of key parameters in this study are very poorly defined and are left as an exercise to the reader/reviewer (eg., NC is, possibly, 'negative control' but that is not written anywhere). For example, Figure 1 should be redone to include information on either the actual dynamic range used, number of PCR cycles done as 'low' in each condition, as well as key informatics steps which are totally absent. The number of PCR cycles in the 'low' condition is of particular importance and should be clearly indicated in a revised figure. The methods for this section should be significantly expanded to include how these cycle numbers were derived.
* One critical parameter, which I am assuming is true, is the downsampling to 2m reads per sample. This is indicated one place in the methods and the antecedent of the sentence is not clear - it is for the whole study or for the fastqc step described. This is perhaps the most important parameter in the study. I am reviewing this manuscript under the assumption that ALL samples are being downsampled to 2m reads for ALL analyses. If that assumption was in any way incorrect, the study should be rejected out of hand.
* The authors describe numerous times that the data below ~7.5ng is of poorer quality and shows more significant artifacts but the authors never indicate that the chemistry used library production specifically has a minimum input range of 10ng. This should be indicated prominently.
* While the authors have done a broad spectrum of dilutions and PCR conditions, it appears each reaction is, itself, a singlicate and could be subject to technical error, for example, errors in serial dilution. The noticeable drop in the graphs of "High" at "7" could be strictly a pipetting error.
* The authors noticeable do not discuss the concept of sequencing saturation. Since the exact same molecules are being sequenced on all 4 sequencers, the same UMIs should be observed. In fact, the most interesting experiment to do would be to subsample to PCR reactions at each time point to look for *loss* of UMI combinations at higher PCR cycles and which genes these UMIs are in.
* The statistics in figure3b are an odd choice and do not accurately reflect the similarity of the analyses. Why this is the one element with statistical analysis is unclear.
* The choice of a UMAP for 3d is perplexing. A simple PCA could elucidate some possible differences. These are not single cell analyses where clusters are expected.

MinorIssues
* The authors indicate that conversion was done prior to sequencing on both the Element AVITI and Singular G4. While these protocols are both available, and the conversion is necessary to remove a possible bias in this study, conversion is not a requirement in general for these sequencers and the associated artifacts observed can be avoided by researchers. This should be indicated prominently in this manuscript as currently described, it could be used as an attack on specific sequencing companies.
* NC is presumably 'negative control' but this is never indicated anywhere in the manuscript. This, and any other unclear labeling should be corrected.
* The text in most of the figures is so small as to be nearly unreadable and figures should be revised to make the text more readable.
* The authors spend extensive time discussing primer dimers withing the Illumina runs that are absent in the Element and Singular runs. They indicate that this is due to extra cleans done by the protocol for Element and Singular. This should be made more prominent as it is critical for interpreting these numbers (the value of the primer-dimer discussion all is questionable).
* The document should be rearranged to focus on the key questions.

**Reviewer 2**

This paper is of broad interest to any lab that routinely makes RNAseq libraries for NGS assessment, or analyzes low input RNAseq datasets. It is of specific interest to Shared Research Resource Facilities that must decide how to best work with RNA from multiple lab sources, species, and input amounts while retaining high-quality data generation capabilities. Methods are appropriate for the conclusions drawn, and conclusions are measured.

This paper could benefit from explicitly describing the number of PCR cycles used for each input rather than the "low, med, high" designations currently being used. While it's understandable that these cycles will change depending on the kit used and to some extent the lab, a more explicit description will help with both replication and advising labs of reasonable starting points when doing their own development.

The 2M read/sample downsampling seems small for a human RNAseq dataset - If the libraries were sequenced to a more "normal" RNAseq depth -25-50M reads/sample, the conclusions could be broadened. For instance: would there be less disparity in duplication rate between the high and low inputs? Conversely, does the duplication in the low-input samples suggest a lack of overall library diversity - do they ever recover the genes that are missing in the current dataset, or are they missing all together?

**Authors’ response**

Reviewer #1:

In this study, Zajac et al. look to investigate the technical impacts of low amounts of RNA input and higher amounts of PCR cycles on RNAseq analysis of gene expression across a number of sequencing platforms. The authors claim that lower amounts of input material result in higher duplicate levels and that UMIs result in more comparable gene expression data. The authors suggest only minimal impact from the choice of sequencing platform.

The question asked by the authors is an important one as many researchers assume these critical parameters are immaterial to their analyses and can focus major scientific conclusions on what were technical artifacts. The authors do observe significant sequencing saturation of samples with lower input amounts and observe negative impacts of additional amplification.

However, the manuscript, as written, is extremely challenging to read and focuses heavily on minor points, hiding the critical results. The figures are poorly designed, do not clearly demonstrate the experiments done, and do not highlight the key results. The study also appears to be highly underpowered with possible technical artifacts visible in the results. For a paper that highlights these artifacts, having an underpowered analysis is not acceptable. Thus, while the issues highlighted by the authors are critical, major revisions should be done prior to acceptance.

Response:We apologize if parts of the manuscript were not as clear as expected, we reorganized the manuscript and rephrased unclear sections, and we respond to the individual issues below.

MajorIssues

* A number of key parameters in this study are very poorly defined and are left as an exercise to the reader/reviewer (eg., NC is, possibly, 'negative control' but that is not written anywhere). For example, Figure 1 should be redone to include information on either the actual dynamic range used, number of PCR cycles done as 'low' in each condition, as well as key informatics steps which are totally absent. The number of PCR cycles in the 'low' condition is of particular importance and should be clearly indicated in a revised figure. The methods for this section should be significantly expanded to include how these cycle numbers were derived.

Response:We add the cycle numbers to the figure, and make the labels clearer. The cycle numbers depend on the input input amount and are chosen to yield approximately the same amount of amplified material for each input concentration. This is in agreement with the protocol that recommends increased cycle numbers for lower input concentrations. We also add the explanation of the bioinformatics workflow to the figure. We also add the following sentence to the beginning of the manuscript: “We generated libraries from human liver RNA at different dilutions (1 ng to 1000 ng) paired with an additional sample of water serving as negative control (NC).”

* One critical parameter, which I am assuming is true, is the downsampling to 2m reads per sample. This is indicated one place in the methods and the antecedent of the sentence is not clear - it is for the whole study or for the fastqc step described. This is perhaps the most important parameter in the study. I am reviewing this manuscript under the assumption that ALL samples are being downsampled to 2m reads for ALL analyses. If that assumption was in any way incorrect, the study should be rejected out of hand.

Response:The assumption is correct, all the samples were downsampled to 2m reads and all the analyses were performed on the subsampled data. Only 4 negative control samples (0 ng) did not have 2m reads. We improve the description: “For each sequencing technology, the data were subsampled to 2,000,000 reads per sample. For the negative control samples, the maximum number of reads was taken if the number of reads was lower than 2,000,000.” and we mention it now also in the results section.

* The authors describe numerous times that the data below ~7.5ng is of poorer quality and shows more significant artifacts but the authors never indicate that the chemistry used library production specifically has a minimum input range of 10ng. This should be indicated prominently.

Response:We made this fact more prominent. In the results section we write: “The libraries were generated with NEBNext Ultra II Directional RNA Library Prep Kit for which the minimal supported input amount is 10 ng.”

* While the authors have done a broad spectrum of dilutions and PCR conditions, it appears each reaction is, itself, a singlicate and could be subject to technical error, for example, errors in serial dilution. The noticeable drop in the graphs of "High" at "7" could be strictly a pipetting error.

Response:For each combination of sample concentration, PCR amplification level, and sequencing technology, we have only one measurement, and thus we can not draw conclusions from individual data points that appear as outliers. However, this design allows us to report on general trends associated with the experimental factors of concentration, amplification, and sequencer. Our statements and their associated statistical significance are focussing on those trends that are observed at multiple sequencers, multiple concentrations or multiple amplifications.

* The authors noticeable do not discuss the concept of sequencing saturation. Since the exact same molecules are being sequenced on all 4 sequencers, the same UMIs should be observed. In fact, the most interesting experiment to do would be to subsample to PCR reactions at each time point to look for *loss* of UMI combinations at higher PCR cycles and which genes these UMIs are in.

Response:To clarify, we created dilutions of the initial total RNA, and then we generated from each dilution a library. The dilutions are done such that we obtained 11 tubes, and each tube would contain an RNA sample with the amount of RNA ranging from 1ng to 1000ng. The RNA in each tube was then amplified and processed to generate a library. For the PCR amplification the number of cycles was chosen according to the recommendations of the NEBnext kit: (1000ng -> 8 cycles, 500ng -> 9 cycles, 250ng -> 10 cycles, ….). This resulted in the data that are labeled as MID in terms of amplification. We additionally processed two additional series of input RNA, once with two cycles less than recommended (LOW amplification), and once with two additional cycles of amplification (HIGH amplification). As a consequence, each of these 33 samples has its own UMI - gene combinations and a potential loss of certain UMI - gene combinations can not be assessed. But what we do see and report in the manuscript is a loss of genes due to the low starting amount and high number of cycles for amplification. The reduced RNA amounts and diversity in the highly diluted samples, and the high levels of amplification (used to compensate for the low starting amount) lead to increased fractions of duplicated reads. However, we do think the comment is valuable and we expand our section on the detected genes. We look at the overlap in the missing genes between sequencers for the low input amounts. We look at the overlap in the missing genes across all low input amounts and we check the gene expression of those genes in the 1000 ng samples We check the overlap in the detected genes between the 4 sequencers at high input amounts

* The statistics in figure3b are an odd choice and do not accurately reflect the similarity of the analyses. Why this is the one element with statistical analysis is unclear.

Response:We do agree with the reviewer that this has not been the best choice of analysis and it does not add any valuable information to the manuscript. We have now indicated that the trend between the input amount/the number of PCR cycles and number of genes is present across all sequencers in figure 3B.

* The choice of a UMAP for 3d is perplexing. A simple PCA could elucidate some possible differences. These are not single cell analyses where clusters are expected.

Response:We have now replaced the UMAP plots with a plot that summarises the contribution of the factors such as the input amount, PCR cycles category and sequencer to the amount of variance in gene counts by fitting a linear (mixed) model. This was the more efficient way of visualising the result that would otherwise require a multiple of PCA plots to elucidate.

Minor Issues

* The authors indicate that conversion was done prior to sequencing on both the Element AVITI and Singular G4. While these protocols are both available, and the conversion is necessary to remove a possible bias in this study, conversion is not a requirement in general for these sequencers and the associated artifacts observed can be avoided by researchers. This should be indicated prominently in this manuscript as currently described, it could be used as an attack on specific sequencing companies.

Response:This has now been indicated in the discussion section of the manuscript where we write “We also investigated the correspondence in the results between the different sequencers. We observe the same patterns of the effect of starting material and the number of PCR cycles on PCR duplicates, similar number of detected genes and a comparable gene expression. We observe only a few minor differences. Firstly, for input amounts below 15 ng, we observe a higher rate of PCR duplicates in data from AVITI and G4, driven by the additional PCR cycles in the conversion protocols. This highlights the increased importance of using UMIs for deduplication or considering sequencer-specific library kits offered from these providers.”

We also mention in the introduction: “There are currently multiple different vendors providing specific library preparations for all aforementioned technologies.”

* NC is presumably 'negative control' but this is never indicated anywhere in the manuscript. This, and any other unclear labeling should be corrected.

Response:We have now indicated NC means negative control in the results section. We generated libraries from human liver RNA at different dilutions (1 ng to 1000 ng) paired with an additional sample of water serving as negative control (NC) and 3 different levels of amplification (categorized as Low, Mid, High, with a 2 cycle difference between consecutive levels), with the number of cycles adjusted to the input amount (New England Biolabs, 2019) (Fig. 1).

* The text in most of the figures is so small as to be nearly unreadable and figures should be revised to make the text more readable.

All figures have been thoroughly revised keeping this in mind.

* The authors spend extensive time discussing primer dimers withing the Illumina runs that are absent in the Element and Singular runs. They indicate that this is due to extra cleans done by the protocol for Element and Singular. This should be made more prominent as it is critical for interpreting these numbers (the value of the primer-dimer discussion all is questionable).

Response:We now rephrase the results section of the manuscript to emphasize the suggested point: “The samples for the different sequencers also differed in read composition. For NovaSeq X and NovaSeq 6000, the proportion of artifactual short reads (<18nt), inferred to be primer dimers, was higher than for the AVITI and G4 sequencers. (Fig. 2a). The percentage ranged from 5.6% to 70.1% for samples of input amounts below 15 ng and from 1.3% to 16.6% for input amounts above 31 ng, with a 10%-25% increase from NovaSeq 6000 to the NovaSeq X. Two samples of input amounts 250 ng and 500 ng, amplified using the highest value of PCR cycles and sequenced with the Illumina sequencers, exhibited a fraction of primer dimers comparable to that of the low input amounts (between 41% - 62%), thus creating outliers. Both the AVITI and G4 exhibited low primer-dimer amounts, which can be attributed to the additional library conversion steps and library cleanup. The percentage of adapter primer dimer contamination for the G4 and AVITI ranged from 0.009% to 3.3% across all input amounts (Fig. 2b).”.

* The document should be rearranged to focus on the key questions.

Response:We now focus on the key points:

Raw Data Quality - different sequencers yield overall very consistent results base quality is high for all sequencers; G4 has slightly more per-base mismatches after alignment that does however not affect the mapping rate negatively

Additional handling of samples results in additional contamination

Among the factors such as input amount, PCR cycles category and type of sequencer, the input amount is the most crucial and influences the percentage of duplicated reads and the number of genes detected:

fraction of reads originating from PCR duplication is minor for input amounts > 125ng but considerable for low inputs and for those low inputs UMI-based deduplication is recommended

Lowly expressed genes get lost from the low input samples

Low input amounts paired with overamplification yield biased gene counts

Reviewer #2:

This paper is of broad interest to any lab that routinely makes RNAseq libraries for NGS assessment, or analyzes low input RNAseq datasets. It is of specific interest to Shared Research Resource Facilities that must decide how to best work with RNA from multiple lab sources, species, and input amounts while retaining high-quality data generation capabilities. Methods are appropriate for the conclusions drawn, and conclusions are measured.

This paper could benefit from explicitly describing the number of PCR cycles used for each input rather than the "low, med, high" designations currently being used. While it's understandable that these cycles will change depending on the kit used and to some extent the lab, a more explicit description will help with both replication and advising labs of reasonable starting points when doing their own development.

Response:We do agree with the reviewer; this is one of the most important parameters in our study.We add the cycle numbers to the figure, and make the labels clearer.

The 2M read/sample downsampling seems small for a human RNAseq dataset - If the libraries were sequenced to a more "normal" RNAseq depth -25-50M reads/sample, the conclusions could be broadened. For instance: would there be less disparity in duplication rate between the high and low inputs? Conversely, does the duplication in the low-input samples suggest a lack of overall library diversity - do they ever recover the genes that are missing in the current dataset, or are they missing all together?

Response:So indeed, the level of duplication at low-input is a consequence of low library diversity. We want to thank the reviewer for the question, we introduced a comment on that in the discussion section. This not only strengthens the context of our study within the broader scientific discussion but also highlights its relevance and contribution to the field. The reason we subsample the data to 2M reads is because for those low input amounts it was not possible to get a higher yield as a result of that. The trends we observe do apply to 25M reads (for those samples where this number of reads can be obtained) as much as they apply to 2M, the only difference we would expect is less disparity between PCR cycle categories and, as the reviewer mentions, an increase in the number of detected genes, which would rather affect biological conclusions for any RNA-seq study rather than technical conclusions that our study focuses on. Subsampling has additionally been shown to produce as accurate results in RNA-seq experiments as using full datasets: Wu et al. 2016.

P. -Y. Wu and M. D. Wang, "The selection of quantification pipelines for Illumina RNA- seq data using a subsampling approach," 2016 IEEE-EMBS International Conference on Biomedical and Health Informatics (BHI), Las Vegas, NV, USA, 2016, pp. 78-81, doi: 10.1109/BHI.2016.7455839

**2^nd^ round**

**Reviewer 1**

The reviewers have addressed my concerns and I recommend the manuscript be published

**Reviewer 2**

The additional information has substantially clarified the manuscript. Two additional changes are recommended below after reading the added material.

pg 9, lines 19-23, authors suggest that missing transcripts from low input amounts (1-15ng) were lost during RNA extraction or depletion during library prep, though they were present in higher input quantities (1000ng at >5 counts). However, this explanation seems inconsistent with the fact that both the 1000, 1 and 15 ng samples were diluted form the same initial RNA pool. However, an additional consistent reading would be that the samples were diluted to a point where the transcript would not necessarily be present in the sample due to either sampling bias or because the sample is lowly expressed. A discussion of the transcript level of the missing genes in the higher input samples would be warranted to address this point.

pg 10, line 2, authors state "technical replicates" and then identify them as samples of the same input quality, but sequenced on different sequencers. These are, by definition, not technical replicates, though the larger point that they could compare UMIs is likely broadly valid, despite the sequencing technique itself likely creating some level of output fragment bias (as noted earlier in the paper).

**Authors’ response**

We would like to thank the reviewers for acknowledging our efforts in the

first part of the revision process. We very much appreciate feedback and additional

improvements.

Reviewer #2:

pg 9, lines 19-23, authors suggest that missing transcripts from low input amounts (1-15ng) were lost during RNA extraction or depletion during library prep, though they were present in higher input quantities (1000ng at >5 counts). However, this explanation seems inconsistent with the fact that both the 1000, 1 and 15 ng samples were diluted form the same initial RNA pool. However, an additional consistent reading would be that the samples were diluted to a point where the transcript would not necessarily be present in the sample due to either sampling bias or because the sample is lowly expressed. A discussion of the transcript level of the missing genes in the higher input samples would be warranted to address this point.

Response: We definitely agree with this and we aim to rectify this inconsistency. We have now revised the sentence to: “This suggests that gene loss primarily results from transcript loss during the serial dilution, driven by either sampling bias or low transcript expression, rather than by sequencing bias.” As is shown in Figure 4D, the genes that are not detected at low levels do have low expression in the 1000 ng samples. We believe that the samples were diluted to low library complexity. The part of the discussion section referring to this phenomenon is: “The impact of those two factors, especially the input amount but also the number of PCR cycles, on the rate of PCR duplicates has already been observed in other studies, both in RNA and DNA sequencing [3,24,25]. The amount of starting material has been inferred to correlate with the library complexity or the number of distinct molecular species in a library [26]. Library complexity has been shown to have a stronger impact on the rate of PCR duplicates in comparison to amplification noise dependent on the number of PCR cycles [26]. Our study is concordant with that conclusion.”

pg 10, line 2, authors state "technical replicates" and then identify them as samples of the same input quality, but sequenced on different sequencers. These are, by definition, not technical replicates, though the larger point that they could compare UMIs is likely broadly valid, despite the sequencing technique itself likely creating some level of output fragment bias (as noted earlier in the paper).

Response: We agree that the term “technical replicates” was misleading and now describe them unambiguously as “samples sequenced on different sequencing machines. The sentence reads now: “UMIs are widely used in the field of RNA-seq to differentiate biological copies from PCR duplicate reads [3] and, in our study, the samples sequenced on the different sequencing machines could be used to test their efficiency in removal of artefacts.”.
